# Professionals’ perspectives on how to address persistent oral health inequality among young children: an exploratory multi-stakeholder analysis in a disadvantaged neighbourhood of Amsterdam, the Netherlands

**DOI:** 10.1186/s12903-022-02510-w

**Published:** 2022-11-14

**Authors:** Awani Balasooriyan, Christine Dedding, Clarissa Calil Bonifácio, Monique H. van der Veen

**Affiliations:** 1 Department of Preventive Dentistry, Academic Centre for Dentistry Amsterdam (ACTA), Gustav Mahlerlaan 3004, 1081 LA Amsterdam, the Netherlands; 2 Department of Ethics, Law and Humanities, Amsterdam University Medical Center (UMC), Amsterdam, the Netherlands; 3 Department of Paediatric Dentistry, Academic Centre for Dentistry Amsterdam (ACTA), Amsterdam, the Netherlands

**Keywords:** Children, Families, Multidisciplinary professionals, Dental Caries, Disadvantaged neighbourhood, Oral Health Inequality, Collaboration

## Abstract

**Background:**

Oral health promotion interventions have had limited success in reaching families in disadvantaged neighbourhoods resulting in persistent oral health inequality. This qualitative study provides insight into professionals’ perspectives on children’s poor oral health (≤ 4 years), their perceptions of the roles and responsibilities, and opportunities for child oral health promotion strategies.

**Methods:**

Thirty-Eight professionals from different domains (community, social welfare, general health, dental care, public health, private sector) working in a disadvantaged neighbourhood in Amsterdam, the Netherlands, participated through 24 semi-structured (group) interviews. Transcripts and notes were analysed through thematic analysis.

**Results:**

Professionals indicate that unhealthy diet, children’s non-compliance, poor parental coping, parental low oral health literacy, parent’s negative attitude, family’s daily struggles, and insufficient emphasis on childhood caries prevention in dental practices, general healthcare and social welfare organisations, underlie poor oral health. They hold parents most responsible for improving young children’s oral health, but recognise that families’ vulnerable living circumstances and lack of social support are important barriers. Interestingly, non-dental professionals acknowledge their beneficial role in child oral health promotion, and dental professionals stress the need for more collaboration.

**Conclusion:**

A broad child-, parental-, and societal-centred educational communication strategy is perceived as promising. Professionals working within and outside the dental sector acknowledge that local and collective action is needed. This involves a better understanding of family’s complex daily reality. Furthermore, intensifying child oral health knowledge in dental practices is essential in collaboration *with* families, general health and social welfare organisations.

**Supplementary information:**

The online version contains supplementary material available at 10.1186/s12903-022-02510-w.

## Introduction

Dental caries is the most common preventable childhood disease worldwide[1, 2]. Millions of young children experience caries in their primary teeth with a globally varying prevalence estimated at 48%, leading to significant impacts on a child’s quality of life and high burden to families and society [3–5]. Caries disproportionally affects disadvantaged groups and represents a disease of socioeconomic and health inequalities [6, 7]. In the Netherlands, young children living in disadvantaged neighbourhoods have an increased risk for dental caries [8]. The prevalence of caries among 5-year-old children from low socioeconomic position (SEP) families is reported as 29%, whereas the prevalence among 5-year-olds in the high SEP group is 19% [9]. Large oral health inequalities were also reported within the low SEP group: caries prevalence among 5-year-olds is 74% when their mothers have a migration background and 22% when their mothers are born in the Netherlands [9]. Early childhood caries levels are predictive of caries levels later in life, and likely of an individual’s overall health [10]. Therefore, it is important to promote and adopt good oral health behaviours from early on, so healthy behaviour becomes a habit.

To tackle the burden of poor oral health in young children, it is necessary to gain a better understanding of the determinants causing oral health inequalities [11, 12]. Previous research has mainly focused on individual-level determinants resulting in behaviour change interventions, such as preventive strategies for oral hygiene and sugar control [8, 13]. However, these preventive approaches have failed to effectively improve oral health behaviour of disadvantaged children [9, 14–17]. To address the persistent oral health inequalities, researchers increasingly acknowledge the importance of social determinants of child oral health, such as family income, educational level, employment status, housing, social support and health status [18–20]. This led to the development of community-based interventions (i.e. oral health education and school-based fluoride application programs) containing high potential to promote oral health of children living in disadvantaged circumstances [12, 21, 22].

Although researchers initially assumed that these community-based interventions would reduce oral health inequalities, recent insights show otherwise [23–26]. Quadri and co-workers (2019) demonstrated that an oral health educational intervention was only effective in children with a higher socioeconomic position [25]. This is in line with a study earlier conducted by Tubert-Jeannin and others (2012), in which an Oral Health Promotion program showed little impact in reducing disparities in oral health [26]. Therefore, the implementation of these interventions may unintentionally contribute to the persistent problem of oral health inequality [12, 15, 27, 28].

The inclusion of the perspectives of local stakeholders in the design and implementation of oral health promotion interventions can be valuable in understanding why community-based interventions disproportionately benefit children growing up in more advantaged families [2, 15, 21]. A successful example of public health intervention is the program Childsmile in Scotland, in which a wide range of stakeholders collaborate to promote child oral health. Its collective approach and implementation of oral health intervention programs in health care, educational and community settings succeeded in improving oral health of young and disadvantaged children [29].

To develop an intervention tailored to the situation in the Netherlands, it is necessary to have more insight into families’ perspectives on child oral health promotion interventions applicable to their daily reality [30, 31]. The same accounts for the perspectives of professionals who regularly work with young children from socially disadvantaged families, such as social welfare workers, health workers, (pre-)school teachers and dental professionals [32]. These professionals are directly or indirectly involved in promoting a healthy lifestyle for vulnerable children and have valuable insights into their living circumstances and unmet (oral) health needs [33]. Professionals’ knowledge can be useful in developing effective oral health promotion interventions targeting disadvantaged families [34]. In addition, it is yet unknown how vulnerable families and stakeholders from different professions think towards a collaborative approach; and if they feel the need to act collaboratively in childhood oral health promotion [35].

The overall aim of this study is to provide insight into the perspectives of professionals regarding poor oral health of young children (≤ 4 years) from families living in a disadvantaged neighbourhood in Amsterdam, the Netherlands, their perceptions of the roles and responsibilities of child oral health, and the opportunities for child oral health promotion strategies. In Amsterdam, approximately 29% of the children did not visit the dentist in 2020, of which the largest group is represented by children aged 4 years or younger [36, 37]. The perspectives of families on child oral health will be investigated in another study. The results may contribute to developing a joint effort between parents and professionals working within and outside the dental sector, aiming to reduce oral health inequalities with a focus on oral health promotion in young children.

## Methodology

### Study design

This qualitative study is nested in a larger Participatory Action Research (PAR) aiming to improve oral health of young children and their parents living in disadvantaged neighbourhoods in Amsterdam, the Netherlands. The focus of this study is Amsterdam New-West. According to the Public Health Service of Amsterdam (GGD Amsterdam), Amsterdam New-West is characterised as a culturally diverse and disadvantaged neighbourhood due to its high scores in childhood obesity, low educational level and poor housing facilities. Children growing up in Amsterdam New-West have an increased risk of experiencing poor oral health [37, 38].

### Recruitment

We used purposive sampling to ensure that the data was gathered from a broad range of professionals, who are knowledgeable in oral health or work on a daily basis with children aged 4 years or younger and their families. We aimed for an equal representation of local stakeholder groups providing services to children and their families living in Amsterdam New-West. We included professionals from various domains, such as community, social and welfare, paramedical health care, general health care, oral health care, public health and the private sector. To identify relevant professionals before the start of the study, we consulted local key informants and experts, such as community workers, academic researchers and municipality advisors regarding social welfare and health.

### Data collection

A semi-structured interview guide was developed based on previous research and initial interviews with key informants (Additional file 1). The interview guide was critically reviewed by MV, CD and CB. Subsequently, the interview guide was optimised in an interview training between AB and residents in paediatric dentistry under the guidance of MV and CB. We asked professionals about their current roles and tasks, their experiences in working with young children and parents, their perspectives on the determinants of poor oral health, their ideas to improve oral health and how they perceive their role in child oral health promotion.

In total, we conducted five semi-structured group interviews ranging from two to six professionals working in the sector of social and welfare (n = 16) and paramedical health care (n = 3) and 19 individual interviews with professionals working in the domains of community (n = 2), social and welfare (n = 5), general and paramedical health care (n = 1), oral health care (n = 9), public health (n = 1) and health insurance (n = 1). The interviews were conducted between June and November 2021 and lasted between 30 and 90 min. AB led the interviews in the Dutch language, which were conducted either online or face-to-face at various locations (i.e. child health clinic, (paediatric) dental practice, general health practice and preschool). With participants’ consent, interviews were either audio-recorded and transcribed (n = 21) or extensive notes were made (n = 3). After each interview, a summary was written consisting of the main topics discussed during the interview and sent back to the respondent for feedback as means of member checking. The transcripts were translated by AB from Dutch to English and were critically reviewed by MV, CD and CB.

### Data analysis

The data were coded with the assistance of the coding software ATLAS.ti. Windows (version 9.0.19.0, Scientific Software Development GmbH in Berlin, Germany). Data analysis followed the six steps of thematic analysis as identified by Braun and Clarke: (1) familiarisation; (2) initial coding; (3) searching for themes; (4) critically reviewing themes; (5) defining themes, and (6) producing the article [39]. A combined inductive and deductive approach was chosen to ensure new themes could be identified. Based on the initial insights, the data was structured using ‘the Conceptual Model on the Influences on Children’s Oral Health’ of Fisher-Owens and colleagues (2007) [18]. This model allows to map the identified themes of factors influencing poor oral health on child level, family level and societal level (see final coding scheme in Additional file 2).

## Results

A total of 38 local professionals shared their perspectives on young children’s oral health in Amsterdam New-West. Four themes were developed, views on (1) Risk factors for poor oral health in young children; (2) Roles and responsibilities in child oral health; (3) Perceived challenges in child oral health care; and (4) Opportunities for child oral health promotion strategies.

### Risk factors for poor oral health in young children

Most professionals are familiar with oral health problems among young children by sharing examples such as “*a mouth full of caries”, “poor oral hygiene”, “black teeth”, “broken teeth”, “rotten teeth”*, and *“children with crowns*”. A preschool teacher experienced children with bad breath by mentioning, *“I also sometimes notice in the morning that children come in, that when they talk to you or come close to you (…) then you think ‘has there been any good oral hygiene before they come here?’”*.

The professionals interviewed consider dietary practices, including unhealthy and high-sugar food consumption, frequent eating moments, prolonged breastfeeding, and long-term bottle use as risk factors underlying poor oral health in young children. Pedagogical employees pointed out that sometimes children are dropped off at preschool or nursery in the morning with candy in their mouths. A paediatric dietician explained, “*It seems as if parents do not know that eating habits, drinking habits and candy habits cause bad teeth*”. A speech therapist referred to parents using “*snacking as an easy means of reward”* for their child. Moreover, the role of grandparents in pleasing with food is highlighted:

“*Grandfathers and grandmothers have great influence. Even though parents do their best at home to provide healthy food and not snack too much, the children are spoiled by grandparents. This, of course, also has a major influence on the teeth”*. (Youth nurse)

Children’s non-compliant and resistant behaviour towards tooth brushing is frequently mentioned as a stumbling block. Professionals illustrated stories of parents such as “*my child doesn’t feel like it”*, “*keeps his mouth shut”*, “*the child doesn’t allow it*”, or “*my child doesn’t want to brush the teeth*”. A youth doctor added: “*Toddlers are quite difficult*”. According to a youth nurse, some parents lack awareness of good oral health hygiene practices and the skills needed to manage their children’s non-compliant behaviour:

“*Parents don’t feel like fighting with their child, and many parents don’t brush well either (…) they just give a toothbrush to their child and then they think, ‘yes, it’s fine’. They also don’t brush it well afterwards (…), and parents also don’t know that until (…) ten or twelve they have to brush afterwards (…) a lot of parents don’t know that either*”.

A majority of the professionals point to parental characteristics as having the most significant influence on children’s poor oral hygiene. Parents are portrayed as unaware and little motivated to establish good oral health habits, assuming that childhood caries is beyond parental control. A paediatric dentist said, “*There are those who just really don’t care. I mean, I had a father this week looking at me, he was like, ‘Yeah, nice of you to say that, but he had one tooth in his mouth himself, and I think he was fine with it”*. A paediatric dietician found “*it is difficult to understand parents, who think it’s normal that their (children) have rotten teeth*”. A youth doctor illustrated how parental low oral health literacy influences their presumption that young children are responsible enough to brush independently by stating:

“*They also don’t know that you just have to brush up to nine years old (...) because they don’t understand that mouth motoric skills are not good yet, they just don’t know that. It is like ‘oh, he can do it himself’ ‘(...) they just don’t know, that knowledge that is missing”*.

Furthermore, it is indicated that parental views towards dental treatments might contribute to poor oral health. A youth nurse explained, “*I think it is not clear to many parents that oral care for children is free*”. Some parents assume that preventive dental checkups are unnecessary for young children, and they only bring their children to the dentist in case of pain:

*“I also have plenty of parents who think ‘well, I only come when I’m in pain’. And then, a child naturally takes over that habit or does not go to the dentist either, because they see that mom or dad don’t do that”*. (Dentist)

Professionals mentioned multiple times that parents feared the high financial costs of oral health care and, therefore, avoided visiting a dental practice. A youth nurse explained, “*Maybe they don’t have money for the dentist, they don’t have good health insurance, because if you don’t have a dentist in the package, it’s all quite pricey. I think that also plays a role*”.

Family household characteristics may also affect oral health in children. A dentist mentioned that large families might be a complicating factor to tooth brushing frequency by stating, “*It’s really hard, if a mother just manages to take care of every child (…) and then she also has to go and brush all those five children at night, they just can’t do it*”. Sometimes parents are aware of the impact of an unhealthy diet. However, daily struggles such as lack of rhythm and structure, stress, agitation, family misery, debts and unemployment complicate children’s oral health. A school dentist explained, “*They know that snacking is not good, and they just don’t manage to apply it in their lives, they have a lot of stress, about work, to make ends meet, they have several children, they just manage to get through the month*”. Besides difficult household conditions, a paediatric dentist mentioned that low social support among vulnerable families might increase the risk for poor oral health:

“* If you see what kind of questions we get at the desk about, ‘there’s a tooth loose, help’. It seems as if they do not have any contact with other people and talk about these kinds of things, because friends also get loose teeth, siblings get it too, and cousins get it too . And yet we are called in a complete panic that a tooth is loose, sometimes I think that those people are kind of very isolated from the whole society*”.

According to the professionals, insufficient emphasis on preventing childhood poor oral health within and outside the dental sector may act as a barrier in child oral health care. A youth doctor claimed that dental practices do not welcome young children by saying: “*Only we sometimes hear that a parent says*, ‘*my dentist doesn’t think it is necessary (…) or come when he is three years old or even’ (…) Some old-fashioned ones say, come on when he’s four*”. A dental prevention assistant indicated that dentists’ focus on prevention instead of curing is still upcoming, but time can be a limitation. A community worker underlined the influence of a dentist, “*I think that there are too few dentists who tell their patient to bring their child from a young age, and then parents think it might not be necessary*”. The lack of awareness regarding child oral health can also be seen among non-oral health professionals, as explained by a dental prevention assistant:

“*My biggest annoyance for years (…) is just the ignorance (…) indeed, the (lack of) awareness among midwives and child health clinics, so really that piece of information, which we provided here, that it has already reached the people in one way or another before they visit here*”.

### Roles and responsibilities in child oral health

Most interviewees highlight that the responsibility for improving young children’s oral health in Amsterdam New-West lies with parents. A dentist noted, “*I try as much as possible to make it clear to the parents that their role is more important than my role*”. A youth doctor added, “*You give (oral health) advice, and yes, it is the responsibility of parents*”. However, parents tend to place responsibility on either the dentist or their child. A dentist illustrated this by saying, *“They (parents) then use me as a bogeyman to raise a child, so to speak, for oral health, like, ‘yes, the dentist is going to tell you how to do it’, while actually, that should be their role*”. A dentist explained that holding parents responsible for a child’s poor oral health might be misplaced considering their lack of awareness about the importance of child oral health:

*“I don’t feel responsible in that sense, but again I do, because I think it is our duty as dentists to inform, repeat, give instructions, treat if necessary, and above all, do a lot of prevention work. We do not see those children every week. And that’s the tricky part, so I guess you can’t point to one person who is responsible for that, because mom and dad can’t do much about it either, because they don’t know”.*

Professionals tend not only to finger-point at parents, but also to other professionals. A general practitioner, for example, mentioned, “*Yes, I look at it now and then, yes, but I think a paediatrician also looks at it, the school doctor also, so those checks are there*”. A dentist advocated for the vital role of other professionals by saying, “*This is a job from the general practitioner, from the school, I think, and all kinds of agencies that kids go to. I think they can play an important role, and if they come here, then we can do the rest here*”. A paediatric dentist would like assistance from public health services (GGD):

“*I would really like help with the children who come to us, extensive help from the GGD, because in most of the families we see, you just do not get there with our help alone because there is just a lot going on more (...) And then the GGD, with their educational capabilities, would be much more suitable to join us in this*”.

Multiple interviewees underpin the importance of a child health clinic considering their broad reach and in-depth knowledge of the household situation of families in Amsterdam New-West. A paediatrician argued:

“*Well, I think this should just be done with the general checkup at the GGD, at the Child health clinic, where the overall care of the child is simply looked at, that should also include the check of the teeth as standard and so direct referral to the paediatric dentist, I think, if necessary*”.

A majority of the non-oral health professionals acknowledge their role in child oral health promotion, and that they could improve their current role. A nursery professional mentioned, “*As professionals, by perhaps just occasionally checking with children what we see and discussing it with parents. (…) or possibly to refer (…) because I think we should take a different, more active role in that*”. A paediatric dietician admitted that awareness of oral health could be enhanced by starting the conversation about oral health with parents. A general practitioner added, “*We pay too little attention to it, I think (…) to the dentist, us too, to bring it up, yes*”. In addition, a speech therapist pointed out that their current practices on oral health could be improved:

“*If I have to be honest, I don’t react much to that, also because this is actually a trigger for me, of, ‘hey, maybe we should pay more attention to that’, I also notice that among colleagues (...) that we notice it, but don’t really take the next step ourselves*”.

Some non-oral health professionals were already providing oral health advice or referring children to the dental practice. A youth doctor indicated, “*Yes, we do advise, I do at least standard at two years, that is actually how we have learned it (…) just to get used to the dentist and get acquainted*”. A youth nurse working at a health centre explained that the topic of oral health is discussed when a child is six months, and parents are helped out in case of problems with brushing. Similarly, the pedagogical workers of a playgroup give oral health-related advice to parents. A paediatric dietician added, “*I now discuss in every conversation that brushing teeth and oral hygiene are very important (…), and I do that every time*”. An employee of a health insurance company referred to their recent action of sending a letter to all low-income families in Amsterdam. This letter explained that oral health care for children under 18 years is free of charge.

### Perceived challenges in child oral health care

Three main challenges are identified based on the perspectives of the professionals. First, providing oral health advice outside the dental sector can be complicated. A speech therapist mentioned, “*If a child comes for language and I suddenly start talking about the teeth, I think that is a more difficult threshold for us to say something about it*”. A youth doctor added, “*Yes, in itself, I think it is a task that belongs to us (…) I am just very curious what would parents want from us? (…) because I don’t know that well*”. Moreover, non-oral health professionals indicate that integrating child oral health into their current working procedures can be challenging. A paediatrician explained this by saying, “*Sometimes it is also quite difficult for us, that you have a certain limited time to do things, and that you can therefore not talk about the teeth for very long, while perhaps you should*”. Two youth doctors illustrated these time constraints:

“*So, as doctors, we have a lot to do in twenty minutes. So questions and attention to oral health care are watering down a bit. We try to quickly look into those teeth (...) And then we just give the advice, because you really don’t have more time for it (...) It’s almost impossible to do, actually*”.

Second, professionals indicate insufficient education in child oral health as a key challenge. Non-dental workers referred to unmet oral health needs in terms of knowledge, skills and availability of oral health materials in multiple languages (i.e. flyers, folders, instructions videos, online tools). Two youth doctors mentioned that child oral health is barely touched upon during their medical education, while they do prefer more training in child oral health:

*Youth doctor 1: “We get [child oral health training] in the [child health clinic] education, but then you have to be in education”.*

*Youth doctor 2: “That is one day”.*

*Youth doctor 1: “And yes, the nurses do not get the [child oral health training], or the doctors who work as youth doctors do not get the [child oral health training] either”.*

*Youth doctor 2: “No, and there is just nothing in the medical education”.*

A youth nurse working for a parenting support organisation opted for a ready-made tool that can be used to provide good oral health advice to parents:

*“Yes, so extra tools that are already ready indeed (…) and made by people with the real expertise, so not from me, with a little basic (oral health) knowledge, but in which a broader vision is unleashed on various problems why a child does not want to brush (…) coming from a multi-problem family”*.

Furthermore, not all dentists are well-trained to treat young children or to provide appropriate oral health messages to parents with young children. A dentist explained, “*Well, maybe for me, I should get more training on how you can transfer this kind of information to parents, to children, so that I can deal with it a little better. And how to have such a conversation, for example, about why such a child has so many cavities*”.

The third challenge includes the lack of communication and collaboration between oral health professionals and non-oral health professionals. A dentist mentioned, “*No, we actually have no contact with the child health clinic (…) we have no contact with school either*”. The importance of effective oral health communication is stressed by a youth doctor, “*I think it’s also a good thing if the dentists are like, ‘Hey, how do we want to get our message across in other ways, besides other professionals, or nice posters or something that you can put up”*. In addition, a general practitioner indicated that communication with surrounding dental practices is limited. A dentist explained that “*personal contact*” with paediatric dentists, other health referrers and neighbouring dentists is unusual by explaining, “*In that sense, we’re kind of a bubble. We maintain our own bubble. And we don’t interfere that much*”. A paediatric dentist underpinned the importance of collaboration with the public health service (GGD):

“*Well, I really want to have good contact with the GGD, that we just have really clear lines, I do have some contact persons, I need to get back to that (...) that we have a kind of collaboration, that all the children we see with caries, just immediately come into the picture of the GGD (...) that we work together in this, I would consider that as important”*.

### Opportunities for child oral health promotion strategies

Key problems of poor oral health in children are structured by Fisher-Owens Model, and proposed opportunities for multidisciplinary professionals are presented in Table 1. A broad child-, parental-, and societal-centred communication strategy was indicated as promising. Informing parents and children about the importance of oral health with a preventive, playful and positive approach may help to increase their awareness and self-efficacy to form healthy oral habits, as explained by a youth nurse:

*“We always try to stimulate the children very much from the positivity (...), so we also reward very much on the competencies that go well, so imagine, a child accepts a toothbrush, then you reward very much on that”.*

Professionals referred to educational events for parents and provision of oral health informative materials (i.e. flyers, posters, videos, online tools, applications) available in different languages containing pictures displaying the importance of brushing from the eruption of a child’s first teeth, the causes and consequences of poor oral health and toothbrush instructions. A paediatric dietician stressed the importance of focusing on the self-efficacy of parents and making them aware of their role in child oral health promotion:

“*Sometimes it is seen as a kind of a fact that my child simply has rotten teeth (...) so those parents do not feel that they can improve on that themselves (...) if you improve your behaviour, that you will simply suffer less from your teeth”*.

Besides, (social) media, campaigns, key figures in the society and religion can be used to effectively transfer oral health information to children and families. A dentist argued that religious leaders could help to effectively disseminate oral health messages by saying, “*So if you use religion, because Islam is actually important here (…) and that is certainly an entrance to reach the people well, because that sticks*”.

**Table 1:** Key problems of poor oral health in children and proposed opportunities according to multidisciplinary professionals

Levels	Key problems as mentioned by professionals	Opportunities as proposed by professionals
Child	Uncooperative and resistant behaviour	Child-centred communication approach in collaboration with parents
	Unhealthy diet	Early prevention
	Poor oral hygiene	Child-friendly oral health materials
	Unpleasant dental care experiences	Self-efficacy of children
Family	Parental beliefs that caries is beyond their control	Parental-centred communication approach
	Limited parental interest in child’s oral health	Increase awareness of child’s oral health
	Lack of parental supervision	Oral health education
	Poor parental coping	Provision of oral health materials
	Unhealthy dietary practices for child	Self-efficacy of parents
	Limited preventive visitations to dental care	Social support (family members)
	Low oral health literacy	Inform parents on the importance of child oral health at different locations
	Daily living conditions	Collaborate with families
	Cultural differences	Take into account family needs
Society - community	Limited community oral health environment	Collaborate with key figures in the community
	Lack of general importance on child oral health	(Social) media campaigns
Society – (non)dental organisations	Current procedures of dental practices, general health care, social and welfare organisations	Increased availability of oral health information throughout neighbourhood and organisations
	Time constraints of current consults of dental practices, general health care, social and welfare organisations	Sense of shared responsibility in child oral health promotion
	Limited knowledge in (child) oral health by non-oral health professionals	Collaborative action among professionals working within and beyond the oral health sector
	Limited skills in effective oral health communication	Setting up new collaborations
	Family’s needs for oral health are unknown	Increase the visibility of child oral health beyond the dental care setting

Most professionals find it helpful to increase the attention given to child oral health beyond the dental setting in order to reach more families. A dentist stressed the importance of collaboration with other healthcare professionals and mentioned, “*I do indeed think that it can help if people also hear from other healthcare providers, from other branches, how important oral health is*”. A community health worker confirmed this by pointing out, “*I think it’s best-appointed by a professional (…) because they (parents) often see it as ‘a doctor tells me so, so it must be true’”*. Parental lack of awareness can be addressed if non-oral health professionals provide information on caries, oral hygiene and free dental care for young children, as noted by a dental prevention assistant:

*“I just think from an earlier point it has to be brought up (...) from the midwife, the child health clinic, because one person here in the chair is not as influential as several people and from several work areas tell you ( ...) And at school, I always think that a lot more attention could be paid to it”*.

Placing informative oral health materials in the waiting room of general health practices, community centres, or other public places might contribute to gaining more attention to child oral health. A dental hygienist advocated that organising educational events at preschools, schools, or child health clinics in collaboration with oral health professionals or dental students could be helpful in establishing a wide reach:

*“I think maybe just from the education of ACTA (Academic Centre of Dentistry of Amsterdam) (…) that the students just go to the schools as part of an internship project (…) to provide oral health education, and you have to leave something behind, (…) because only providing oral health education is pointless, so oral health education must be given to the children and to the teacher (…), and you have to deliver something, just like a game, or a poster or a folder, that the children have to give this to their parents, that it will continue in that way”.*

Although most professionals perceive it as challenging to change oral health behaviour, they unanimously agreed that better interdisciplinary collaboration between parents, the community and professionals working within and beyond the dental sector play an essential role in child oral health promotion. Most of the interviewees advocated that it is vital to approach parents with a manner of positivity, empathy and openness:

*“What I experience is what they (parents) like, is if you really ask or talk about their culture (...) their environment, that it is not like I am the professional, you come here, and this is our only relationship (...) they really enjoy being able to tell something about their own life now and then (…), so you also build a certain bond to that extent”* (Speech therapist).

Finally, the essence of setting up new collaborations between different professions such as child nurseries, preschool and school teachers, child health clinics, dental health schools, community centres, paediatric dietary practices, paediatric dental practices, obstetrics practices, general health practices, hospitals, paramedical practices, health insurance companies, government, religious organisations and dental practices, was highlighted.

## Discussion

This study is one of the first to shed light on multidisciplinary professionals’ perspectives within and outside the dental sector regarding the causes of poor health of young children (≤ 4 years) from families living in a disadvantaged neighbourhood, their perceptions of the roles and responsibilities, and their ideas concerning child oral health promotion strategies.

In summary, most professionals describe that poor oral health in young children is primarily affected by unhealthy diet, children’s non-compliance towards brushing and lack of skills in parents to manage these, parental low oral health literacy, parents’ negative attitude towards poor oral health and family’s daily struggles. Besides, insufficient attention on preventing poor oral health in current work practices of dental practices, general health and social welfare organisations are considered risk factors. Parents are held most responsible for improving young children’s oral health, but most professionals recognise that vulnerable families’ living circumstances and lack of social support are important barriers. Interestingly, non-dental professionals acknowledge their beneficial role in child oral health promotion, and dental professionals stress the need for more collaboration. However, setting up local and collaborative action among families, the community and professionals is accompanied by obstacles, such as approaching and motivating families, integrating child oral health in the non-dental sector, and insufficient education on the importance of and how to prevent poor oral health.

In scientific research, it is still common to portray socially disadvantaged populations as ‘unhealthy’ and ‘problematic’ with inherently ‘deviant’ personality traits and ‘adverse’ culture [40]. Although scientific evidence clearly shows that children from vulnerable families have an increased risk for poor oral health, it is unjust to presume that these families are simply not capable to adhere to preventive caries measures [8]. It should be taken into account that the living environment contributes to an unhealthy lifestyle in vulnerable communities. We have to be cautious in unintentionally stigmatising socially disadvantageous as ‘problematic’, ‘unhealthy’ or ‘hard-to-reach’. We should consider their individual oral health needs in relation to the daily context and challenges they face, and how well-equipped professionals are in providing good care to these families. Therefore, working together with families and taking into account their surrounding social and contextual factors allow us to better understand the complex reality of vulnerable families. This approach might be highly beneficial in combating oral health inequalities among young children [33, 41].

A family-centred educational communication strategy was perceived as promising by most professionals implying that vulnerable families should change their oral health behaviour. These so-called victim-blaming strategies assume that knowledge and skills automatically lead to behavioural change [15]. However, according to Kwan and Peterson (2010), individualised education strategies may have a limited impact on families living in disadvantaged circumstances, since the wider social determinants of poor oral health in young children are not accounted for. Children growing up in more privileged households may benefit from informative oral health approaches even more than those in socially disadvantaged groups. This could, unintentionally, widen oral health inequality among young children [15, 42]. Moreover, Albino and Tiwari (2019) criticised such educational interventions for focusing solely on providing oral health information to parents without considering their family circumstances [13]. Solely a child oral health promotion intervention focusing on education and knowledge will most likely not lead to sustainable behaviour change in children and their families. To overcome this, dental and non-dental professionals should critically reflect on their own actions and how they can use their position to address the root causes of poor oral health in young children and persistent oral health inequalities.

To date, it is not very common for preschool and school teachers, general health workers and social welfare workers to raise awareness of promoting oral health among parents. Non-oral health professionals interviewed tend to stick within their expertise area and feel not comfortable enough to provide oral health messages to parents. Interestingly, dental health workers indicate encountering difficulties in combining oral health education with nutritional and psychological advice. According to Wagner and Heinrich-Weltzien (2017), setting up an interdisciplinary collaboration is an effective approach to preventing caries in children aged five years [43]. Blomma and Krevers (2020) concluded that successfully implementing an interdisciplinary public preventive oral health project requires a shared common view by the involved disciplines and professions on how to collectively prevent childhood caries among children living in disadvantaged areas [35]. Bhatti and colleagues (2022) advocated that the implementation of a complex oral health intervention targeting parents with infants (9–12 months) requires increased local collaboration and communication between health visitors and dental practices [44]. The projects ‘Healthy Toddler Mouths’ and ‘Giga Whole’ are promising initiatives aiming for a stronger interdisciplinary approach to improve oral health care of young children in the Netherlands [32, 45]. Inspired by the aforementioned studies, setting up a similar collaborative approach seems most promising when starting on a small scale. First, it is needed to facilitate local networks between dental practices, general health and social welfare organisations. Second, support is necessary for multidisciplinary professionals in providing oral health education combined with general health advice. Third, it is important to avoid conflicting information about providing (oral) health habits by establishing a general consensus on (oral) health information. Most importantly, it is vital to consider the role of the community, the supporting families, child caregivers and friends in child oral health promotion [41].

The challenge is to increase awareness of preventing poor oral health in childhood in a sustainable manner. Besides, it should be possible for vulnerable families to include oral hygiene habits in their daily routine. For future research, it is recommended to further explore the perspectives on child oral health among families living in a disadvantaged neighbourhood. In addition, more research is needed on how to reach disadvantaged families with appropriate childhood oral health promotion programs. It is also recommended to conduct a similar study in another population group to better understand poor oral health in young children from different target populations. This in-depth knowledge will be beneficial in developing a population-specific oral health promotion intervention. Finally, it will be beneficial to make a first attempt in creating new collaborations between local professionals aiming to provide (oral) health care equally for *all* young children.

This study is limited in terms of its representation of all professionals involved in children’s first four years of life. Most participating professionals work in the domains of oral health, general health and social welfare organisations, while professions related to obstetrics, maternity care, child physiotherapy, toddler sports clubs, municipality and health insurance were not well represented. Arguments such as “*Child oral health is beyond our scope*” or “*Oral health advice is already included in our current consults with (future) parents*” were frequently mentioned. Another limitation inherent to the qualitative study design is the lack of generalisability of the findings. A strength of the study is that the importance of child oral health among local professionals has gained renewed attention. Through participation in this study, professionals working beyond the oral health sector recognised their potential beneficial influence and the need to increase collaboration with dental professionals.

## Conclusion

In summary, promoting good oral health habits in vulnerable families is a great challenge. Especially since most professionals recognise that these families’ living circumstances and lack of social support are complicating factors. A broad child-, parental-, and societal-centred educational communication strategy is perceived as promising. Professionals working within and outside the dental sector acknowledge that *broad, local and collective* action is needed to tackle persistent oral health inequality among young children. Such interdisciplinary collaboration requires a better understanding of family’s oral health needs as well as addressing the needs of professionals working within and outside the dental care setting. These insights can be used to develop an appropriate child oral health promotion intervention meeting family’s needs and circumstances.

## Electronic supplementary material

Below is the link to the electronic supplementary material.

**Additional file 1.** Interview guide

**Additional file 2.** Final coding scheme

## Data Availability

The data that support the findings of this study are available from the corresponding author upon reasonable request.
