# Supplementary material for: Professionals’ perspectives on how to address persistent oral health inequality among young children: an exploratory multi-stakeholder analysis in a disadvantaged neighbourhood of Amsterdam, the Netherlands
Source: BMC Oral Health. 2022 Nov 14;22:488. doi: 10.1186/s12903-022-02510-w (PMC9664661; doi:10.1186/s12903-022-02510-w)
Supplement: Supplementary file 1 — Additional file 1. Interview guide [file 12903_2022_2510_MOESM1_ESM.docx]

Additional File 1: Interview guide for professionals

**INTRODUCTION**

1. Can you describe the current work procedures, tasks or duties of a [name profession]?

FOLLOW-UP QUESTIONS

- What is your professional background?
- What kind of work do you specifically do?

**MOTIVATION FOR PARTICIPATION IN THE STUDY**

2. Can you explain your motivation to participate in this study?

FOLLOW-UP QUESTIONS

- Why is the topic of child oral health important to you?
- How does oral health play a role in your current work?
- What information on child oral health is currently provided by you or your colleagues?
- Is the topic of child oral health currently being discussed in your consultations with parents?
- Can you explain why oral health is currently not addressed in your consultations?
- Do you ever get oral health-related questions from parents?

**EXPERIENCES IN WORKING WITH FAMILIES IN A DISADVANTAGED NEIGHBOURHOOD**

3. This study focuses on improving the oral health of young children (0-4 years) from families with a low socioeconomic position (SEP) in Amsterdam New West. We see that these children suffer more from cavities than those who grow up in richer environments. Can you tell us about your experiences in working with these children and their parents?

FOLLOW-UP QUESTIONS

- What is it like to work in this neighbourhood?
- How do you do that exactly?
- Can you provide an example?
- What do you find difficult?
- Would you like support?

**GENERAL HEALTH STATUS OF YOUNG CHILDREN FROM FAMILIES WITH A LOW SEP**

4. Before discussing the oral health situation of children (0-4 years) from low SEP families in New West, I would like to get a better idea of the general health situation of these children. What health-related problems do you see among young children from low SEP families in this neighbourhood?

FOLLOW-UP QUESTIONS

- What health problems stand out?
- What do you think could be the causes of these health problems?
- How do parents deal with these health problems?
- How are these health problems currently being addressed?
- What is your role in dealing with these problems?
- Who or what do you think could help solve these problems?
- Do you have insight into how these problems are dealt with, for example, by neighbourhood initiatives?

**ORAL HEALTH SITUATION OF YOUNG CHILDREN FROM LOW SEP FAMILIES**

5. Let's continue with the oral health situation of young children from low SEP families in New West. As I mentioned earlier, these children are more prone to cavities. Can you explain if this is something you recognise from your own practical experiences?

FOLLOW-UP QUESTIONS

- Can you describe what you see regarding the high prevalence of dental caries in young children? (target group?; age?)
- What problems do you see with the oral health of young children?
- What do you think could be the causes of these oral health problems?
- If you see a child with bad teeth, how do you deal with this? (do you give advice?; do you refer to the dentist?; how do parents react to this?)
- How do parents deal with these oral health problems?
- Do you have any idea why you are not familiar with the high prevalence of caries among young children in this neighbourhood?

**ADDRESSING ORAL HEALTH ISSUES AMONG YOUNG CHILDREN FROM LOW SEP FAMILIES**

6. Now, we come to the last point that I would like to discuss with you, namely what approach is needed to improve the current oral health situation of these vulnerable children in New-West. Do you have any ideas on this?

FOLLOW-UP QUESTIONS

- What would be necessary to address the oral health problems among young children in Amsterdam New West?
- How could we reach and involve this target group in our research? What approach is needed for this?
- What needs do you have?
- What wishes do you have?
- What could be possible solutions?
- Who or what must change?
- Who or which parties in the neighbourhood could help?
- Who or which parties are already working on solving this problem?
- How can we help you?
- Could we work together to tackle this problem?
- Do you have any ideas on how we can better promote the importance of oral health for young children?

**CLOSING REMARKS**

We have reached the end of our conversation. Thank you for your time and for sharing all the information. Are there any issues we haven't discussed yet? In case of any additional questions, can I approach you about this?
