# Supplementary material for: Professionals’ perspectives on how to address persistent oral health inequality among young children: an exploratory multi-stakeholder analysis in a disadvantaged neighbourhood of Amsterdam, the Netherlands
Source: BMC Oral Health. 2022 Nov 14;22:488. doi: 10.1186/s12903-022-02510-w (PMC9664661; doi:10.1186/s12903-022-02510-w)
Supplement: Supplementary file 2 — Additional file 2. Final coding scheme [file 12903_2022_2510_MOESM2_ESM.docx]

Additional File 2: Final coding scheme

| **Themes** | **Categories** | **Sub-categories** |
| --- | --- | --- |
| 1. Risk factors of poor oral health | General awareness | - Familiarity with problem - Unfamiliarity with problem |
| 1a. Risk factors of poor oral health – Child level | Behaviour | - Non-compliance - Non-cooperative - Resistance towards brushing - Less approachable |
|  | Personality traits | - Autism - Hereditary - Motoric skills |
|  | Diet | - Unhealthy food - Bottle use - Prolonged breastfeeding - Frequent eating moments |
|  | Dental health care experiences | - Dental trauma - Perception of the dentist as a bogeyman - Dental fear - Unpleasant dental treatments |
| 1b. Risk factors of poor oral health – Parents / Family level | Parental attitude | - Conscious, responsible and concerned (positive attitude) - Limited interest, little motivation or child oral health is perceived as unimportant - Feelings/beliefs - Difficult to approach |
|  | Parental behaviour | - Parental supervision - Parenting skills - Dietary practices - Dental health care experiences |
|  | Parental knowledge | - Low oral health literacy - Limited knowledge of healthy diet for children |
|  | Social support | - Role of grandparents (limited knowledge and tend to spoil grandchildren) - Social isolation |
|  | Family household characteristics | - Family composition - Family functioning - House conditions - Daily struggles - Finances - Socioeconomic position - Culture |
| 1c. Risk factors of poor oral health - Society (Community) | Attitude | - Limited interest or little motivation - Perceived differences between generations |
|  | Behaviour | - Poor oral hygiene |
|  | Knowledge | - Limited oral health information in the community - Many oral health-related questions - Ignorance of people |
|  | Access to dental health care | - Barriers: migration background, unknown, fear of high costs, shame - Dental care is not very likeable |
|  | Community oral health environment | - Cheap and unhealthy food - No attention to oral health on TV - Oral health has a lower priority |
|  | The general importance of child oral health | - Limited importance of oral health - Limited focus on oral health during the education of non-oral health professions - Limited information on oral health in health booklet disseminated outside the dental sector |
| 1d. Determinants of poor oral health - Society (Organisations) | Child daycare  Child health clinic  Church  Community worker  Dental practice  General practice  Health insurance  Municipality  Parenting support  Paediatrician  Paediatric dentist  Paediatric dietician  Playgroup  Preschool  School dentist  Speech therapy | - Current work procedures of general dental care practices - Current work procedures of non-dental care practices - Time constraints - Limited affinity with children - Different scope - Limited knowledge, skills, training, and education in child oral health |
| 2. Roles & Responsibilities | Child daycare  Child health clinic  Church  Community worker  Dental practice  General practice  Health ambassador  Health insurance  Municipality  Parenting support  Paediatrician  Paediatric dentist  Paediatric dietician  Playgroup  Preschool  School dentist  Speech therapy | - Role of parents - Finger-pointing between professionals - Non-oral health professionals acknowledge their role in child oral health promotion - Oral health advice already provided by non-oral health professionals - Multidisciplinary collaboration |
| 3a. Challenges and unmet needs | Parents / Family | - Unknown needs of parents - Parental characteristics - Family living conditions |
| 3b. Challenges and unmet needs | Professionals (society) | - Perceived difficulties in providing oral health advice outside the dental sector - Time constraints - Insufficient education in child oral health - Lack of communication and collaboration between oral health professionals and non-oral health professionals |
| 4a. Opportunities – Child level | Child-centred approach | - Positive - Self-efficacy - Playful - Educational - Focus on early prevention - Child-friendly materials |
| 4b. Opportunities – Parents / Family level | Parental-centred approach | - Informative - Playful - Collaborative - Positive - Bond of trust - Use of professionals - Low key and stepwise - Active and in person - Increase visibility - Increase awareness of oral health |
|  | Education | - Educational events at schools/playgroups - Oral health materials |
|  | Self-efficacy parents | - Parenting skills - Make parents aware that they can change oral health behaviour - Focus on beneficial influence of parents |
|  | Social support | - Role of grandparents - Siblings |
| 4c. Opportunities – Society (Community) | Key figure | - Religion - influencer |
|  | Media | - Campaign |
| 4d. Potential solutions – Society (Professionals) | Multiple information sources on oral health throughout the neighbourhood | - Oral health information is disseminated through the dental and non-dental sector - Combined health advice |
|  | Shared-responsibility professionals | - Acknowledgement of shared effort in child oral health promotion |
|  | Ideas for collaborative actions | - Posters at the bus stop - Educational sessions at different locations - Oral health information at stores - Child brushing week |
|  | Collaboration partners | - Oral health professionals - Non-oral health professionals |
